# Isolation of the human-associated *bla*_CTX-M-15_-harbouring *Klebsiella pneumoniae* ST307 from a tortoise in the UK

**DOI:** 10.1099/acmi.0.000172

**Published:** 2020-09-24

**Authors:** Geoffrey Foster, Manal AbuOun, Romain Pizzi, Bryn Tennant, Margaret McCall, Muna F. Anjum

**Affiliations:** ^1^​ SRUC Veterinary Services, Inverness, IV2 5AN, UK; ^2^​ Animal and Plant Health Agency, New Haw, Surrey, KT15 3NB, UK; ^3^​ Zoological Medicine Ltd, 40 Charlton Grove, Roslin, EH25 9NX, UK; ^4^​ SRUC Veterinary Services, Penicuik, Midlothian, EH26 0PZ, UK

**Keywords:** *Klebsiella pneumoniae*, ST307, tortoise, multidrug resistant, CTX-M-15

## Abstract

The ST307 multidrug-resistant CTX-M-15-producing *Klebsiella pneumoniae* is an emerging pathogen, which has become disseminated worldwide in humans but is rarely reported from other reservoirs. We report the first isolation of *K. pneumoniae* from an animal in Europe and also from a reptile, a captive tortoise, whose death it probably caused. Detection of this clone from an animal adds to evidence of niche expansion in non-human environments, where it may amplify, recycle and become of greater public health concern.

## Background

The CTX-M-15-producing *Klebsiella pneumoniae* ST307 has emerged globally in humans, being associated with sepsis, pneumonia and urinary tract infections caused by extended-spectrum β-lactamase (ESBL)- and carbapenemase-producing *K. pneumoniae* that can be multi- or extensively drug-resistant, and hence difficult to treat [1–3]. Amongst animals, *K. pneumoniae* ST307 is rare but the animal niche may be expanding. Previously, it has been identified from a dog in Brazil [4] and from 1 of 29 rats in Africa that were trapped and tested for ESBL-producing *Enterobacteriaceae* [5]. We wish to report the identification of *K. pneumoniae* ST307 in a captive African spurred tortoise (*Centrochelys sulcata*) in a zoological park in the UK.

## Case presentation

Four tortoises had been captive in a zoological park following seizure by customs upon entering the UK in 2013. They were kept in an enclosure that is separate from other animals in the zoo and there had been no previous apparent illness or treatment of any of the four tortoises during their time at the zoo. Keepers always wore disposable gloves for handling the tortoises and they were not handled by members of the public. Two of the tortoises had been under treatment for lethargy, inappetence and respiratory signs. Both were treated with 5 mg kg^−1^ injectable 2.5 % enrofloxacin every second day from 29 April and were tube-fed. They were kept in separate hospital tortoise boxes, enclosed indoors with separate heating and UVb lighting, medicated individually, given warm water baths daily and tube-fed if not eating.

## Investigation

One of the sick tortoises died 28 May 2019 and was subject to a post-mortem examination at SRUC Veterinary Services Edinburgh within 24 h. Gross pathology diagnosis was pneumonia, and lung tissue was selected for culture on Columbia sheep blood agar, MacConkey agar without salt and Sabouraud dextrose agar with chloramphenicol (Oxoid, Perth, UK). A confluent growth of *K. pneumoniae* and a mixed fungal growth of *Geotrichium candidum* and *Penicillium* sp. were obtained.

**Fig. 1.**
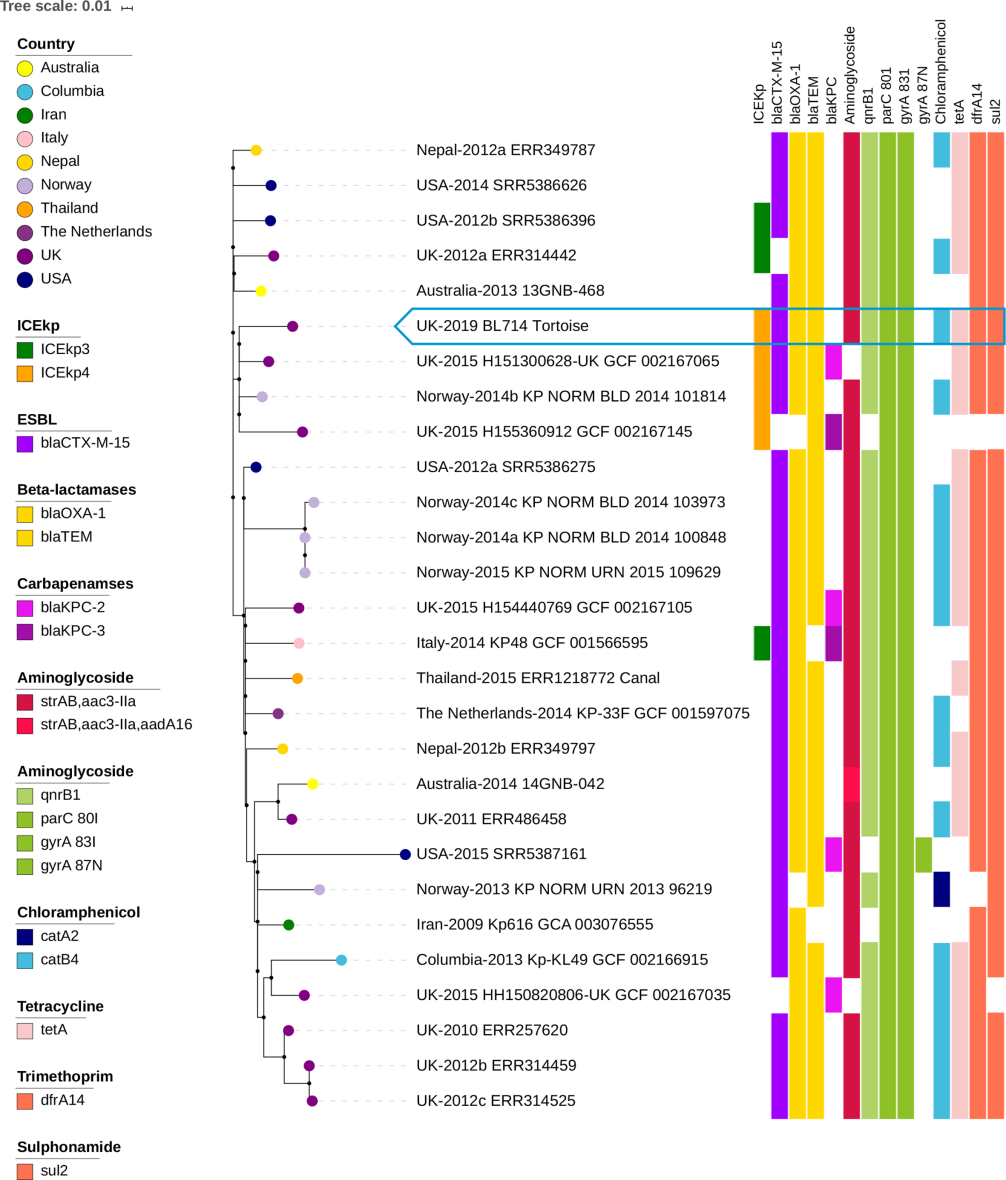
Phylogenetic tree of *K. pneumoniae* ST307 isolates. A recombination-free core genome SNP phylogenetic tree constructed using the oldest *K. pneumoniae* ST307 isolate from Iran in 2009 as the reference. A selection of available *K. pneumoniae* ST307 genomes and the *K. pneumoniae* ST307 isolated from the tortoise were included. The ICE*Kp* element, which carries the yersiniabactin siderophore genes, and AMR genes harboured by isolates are represented by colour blocks.

DNA was extracted from the *K. pneumoniae* isolated from lung tissue and whole-genome sequencing (WGS) was performed using Illumina NextSeq for detailed molecular characterization; the WGS results were submitted to ENA under PRJEB36913. Species classification by Kraken [6], confirmed that the species with the highest match was *K. pneumoniae* and *in silico* multilocus sequence typing [7] indicated the isolate to be of sequence type (ST) 307 (ST307). A recombination-free core genome single-nucleotide polymorphism (SNP)-based phylogenetic tree was constructed with the tortoise isolate (BL714) and 27 previously identified global *K. pneumoniae* ST307 isolates [2]. Isolate BL714 was phylogenetically closest to a UK human clinical isolate from 2015 (UK-2015_H151300628-UK_GCF_002167065; Fig. 1), with 53 SNP differences between the tortoise isolate and the 2015 human UK isolate. The Kleborate tool (https://github.com/katholt/Kleborate) was used to identify presence of known virulence factors associated with hypervirulence and invasive disease in human infections [8, 9]. Only the yersiniabactin siderophore gene cluster within the conjugative element ICEKp4 was present. This element has been reported from a minority of ST307 isolates previously [2] and the tortoise isolate BL714 in fact clustered with other isolates harbouring the ICEKp4 element (Fig. 1). The aerobactin and salmochelin siderophore gene clusters, and the well-characterised genotoxin colibactin virulence determinant, were all absent. Similar to other ST307 clones, BL714 was of capsular type KL102 (KN2).

Antimicrobial sensitivity testing of the *K. pneumoniae* isolate, which was performed by disc diffusion according to the European Committee on Antimicrobial Susceptibility Testing (EUCAST) methodology and breakpoints, and confirmed by genotypes predicted from WGS using the APHA SeqFinder pipeline [10], indicated the isolate to be multidrug-resistant (MDR). Antimicrobial resistance (AMR) was recorded for ampicillin, amoxicillin/clavulanate, piperacillin/tazobactam, cefotaxime, ceftazidime, cefpodoxime, cephalexin, cefovecin, ciprofloxacin, enrofloxacin, gentamicin, tetracycline, sulphamethoxazole/trimethoprim and chloramphenicol. Sensitivity was only detected for ertapenem and neomycin. The AMR gene for resistance to ESBLs was *bla*_CTX-M-15_; for beta-lactamases it was *bla*_TEM-1b_ *, bla*_OXA-1_; for gentamicin it was *aac3-IIa*; for ciprofloxacin/enrofloxacin it was *qnrB1*; for tetracycline it was *tet(A)*; for sulphamethoxazole/trimethoprim they were *sul2*, *dfrA14*; and for chloramphenicol it was *catB4*. The AMR profile of the tortoise isolate was the same as that for a Norwegian human isolate (Norway-2014b_KP_NORM_BLD_2014_101814) that was present within the same sub-cluster, but different from those of the two human UK isolates also in this sub-cluster, which harboured variants of carbapenem resistance genes (*bla*_KPC2_ and *bla*_KPC3_; Fig. 1).

## Discussion

To our knowledge, this is the first report of the isolation of the global human ESBL-producing MDR ST307 *K. pneumoniae* clone from an animal in Europe. It is also the first report of its isolation from a reptile globally, where it likely played a part in the development of pneumonia, as has been reported for humans [3], and the subsequent death of the animal. Nevertheless, the ST307 clone has only rarely been detected from non-human sources such as companion animals [4] and environmental/sewage water samples [2], and so this detection represents an expansion of its presence in a non-human niche. Detection in a zoo animal is also notable, as it suggests the possibility of anthropogenic transmission. Furthermore, our findings draw attention to the potential role of captive exotic animals in the global dissemination of a human pathogen that is MDR.

Although the group of tortoises in the premises in this study were not handled by the public, other zoos do encourage interactions with tortoises. A recent study of animals in petting zoos in Israel recovered ESBL-producing *Enterobacteriaceae* from the surface and faeces of a number of animals, including *Enterobacter cloacae* ST1152 from the faeces of one of two tortoises tested, an African spurred tortoise (*Centrochelys sulcata*) [11]. While considerable focus is placed upon the antimicrobial treatment and stewardship of humans and domesticated animals, the role of wild and exotic animals in the dissemination of antimicrobial resistance, especially in zoonotic pathogens of public health concern, continues to receive relatively minor attention. This paper suggests that monitoring of wildlife for both AMR and important human pathogens is a gap that should be considered, if niche expansion and recycling of highly successful human MDR clones, such as *Klebsiella pneumoniae* ST307, which may also harbour carbapenemase resistance, are to be avoided in future, whether in human or other environments.
